# Supplementary material for: Genome-wide association studies meta-analysis uncovers NOJO and SGS3 novel genes involved in Arabidopsis thaliana primary root development and plasticity
Source: Mol Biol Rep. 2024 Jun 14;51(1):763. doi: 10.1007/s11033-024-09623-1 (PMC11178574; doi:10.1007/s11033-024-09623-1)
Supplement: Supplementary file 1 — Supplementary Material 1 [file 11033_2024_9623_MOESM1_ESM.docx]

**Table S1. Growth conditions and number of accessions used in each study**

| Study | Abreviation | Days after germination (DAG) or sowing (DAS) evaluated | Number of acccessions | Growth conditions |
| --- | --- | --- | --- | --- |
| Lachowiec, et al., 2015 | A7 | 7 DAS | 93 | MS 1x basal salt medium supplemented with 1× MS vitamins, 0.05%MES (wt/vol), and 0.3% (wt/vol) phytage in darkness |
| Julkowska et al., 2017 | B8 | 8 DAS | 347 | MS and Skoog medium 0.5x, 0.5% sucrose, 0.1% MES monohydrate, and 1% Daishin agar, pH 5.8 (KOH). Long-day conditions (21°C, 70% humidity, 16/8-h light/dark cycle) |
| Ristova et al., 2018 | C10 | 10 DAS | 192 | One‐fifth‐strength MS medium with MES buffer, 1% sucrose, 0.8% agar, and adjusted to pH 5.7. Long‐day conditions (21°C, 16 h light/8 h dark cycle) |
| Bouain et al., 2018 | D1-D7 | 1-7 DAG | 231 | 1X MS-agar medium: 0.5 mM KNO3, 1 mM MgSO4, 1 mM KH2PO4, 0.25 mM Ca(NO3)2, l00 μM NaFeEDTA, 30 μM H3BO3, l0 μM MnCl2, l μM CuCl2, 15 μM ZnSO4, 0.1 μM (NH4)6Mo7O24, and 50 μM KCl, in presence of 1% (wt/vol) sucrose and 0.8% (wt/vol) agar. 22°C long-day conditions (16 h light, 8 h dark) |
| Bouain et al., 2019 | E4,E5 | 4,5 DAG | 227 | 1X MS-agar medium, which contained 1 mM KH2PO4, 1 mM MgSO4, 0.5 mM KNO3, 0.25 mM Ca(NO3)2, 10 μM MnCl2, 30 μM H3BO3, 1 μM CuCl2, 0.1 μM (NH4)6Mo7O24, 50μM KCl, 100 μM NaFeEDTA and 15 μM ZnSO4 in presence of 0.8% (wt/vol) agar and 1% (wt/vol) sucrose. Plants were grown at 22˚C, long-day: 8 h dark, 16 h light). |
| Li et al., 2019 | F3-F8, F10, F13 | 3-8, 10, 13 DAS | 319 | 1× MS with 100 µM Fe (III)-EDTA and 1 % agar. 16/8-h light-dark cycle at 21 °C |
| Ogura et al., 2019 | G5 | 5 DAG | 123 | MS 1x, 1% sucrose, 0.8% agar, pH 5.6. 16 hours of light conditions, 21 °C |
| Justamante et al., 2019 | H6 | 6 DAS | 120 | 75 mL of one-half- MS medium with 2% sucrose, 8 g/L plant agar and 1× Gamborg B5 vitamin mixture. 22 ± 1 ◦C during 3 days in a nearly vertical position. Plates were unwrapped (3 days after sowing) and grew during another 3 days with continuous light (50 µmol·m−2 ·s −1 |
| Deolu-Ajayi et al., 2019 | I6-I9 | 6-9 | 33 | MS 0.5 with vitamins, 0.5% sucrose, 0.1% MES Monohydrate, pH 5.8 with KOH, and 1% agar. 20°C, 12/ 12 hours light/ dark, 122 µmolm-2s-1 light intensity, and 70% Relative Humidity |
