# Supplementary material for: Genome-wide association studies meta-analysis uncovers NOJO and SGS3 novel genes involved in Arabidopsis thaliana primary root development and plasticity
Source: Mol Biol Rep. 2024 Jun 14;51(1):763. doi: 10.1007/s11033-024-09623-1 (PMC11178574; doi:10.1007/s11033-024-09623-1)
Supplement: Supplementary file 5 — Supplementary Material 5 [file 11033_2024_9623_MOESM5_ESM.docx]

**Table S5. Shapiro-wilk test of normality**

| **Shapiro-Wilk test** | **A7** | **B8** | **C10** | **D1** | **D2** | **D3** | **D4** | **D5** |
| --- | --- | --- | --- | --- | --- | --- | --- | --- |
| W | 0.9826 | 0.9948 | 0.9907 | 0.9785 | 0.9924 | 0.9950 | 0.9962 | 0.9963 |
| P value | 0.2599 | 0.2888 | 0.2611 | 0.0015 | 0.2815 | 0.6599 | 0.8499 | 0.8696 |
| Passed normality test (alpha=0.05)? | Yes | Yes | Yes | No | Yes | Yes | Yes | Yes |
| P value summary | ns | ns | ns | ** | ns | ns | ns | ns |

| **Shapiro-Wilk test** | **D6** | **D7** | **E4** | **E5** | **F3** | **F5** | **F8** | **F10** |
| --- | --- | --- | --- | --- | --- | --- | --- | --- |
| W | 0.9960 | 0.9963 | 0.9885 | 0.9925 | 0.9685 | 0.9949 | 0.9961 | 0.9915 |
| P value | 0.8129 | 0.8696 | 0.0694 | 0.3168 | <0.0001 | 0.3710 | 0.6267 | 0.0651 |
| Passed normality test (alpha=0.05)? | Yes | Yes | Yes | Yes | No | Yes | Yes | Yes |
| P value summary | ns | ns | ns | ns | **** | ns | ns | ns |

| **Shapiro-Wilk test** | **F13** | **G5** | **H6** | **I6** | **I7** | **I8** | **I9** |
| --- | --- | --- | --- | --- | --- | --- | --- |
| W | 0.9942 | 0.9925 | 0.9896 | 0.9701 | 0.9710 | 0.9689 | 0.9868 |
| P value | 0.2718 | 0.7624 | 0.4964 | <0.0001 | <0.0001 | <0.0001 | 0.0063 |
| Passed normality test (alpha=0.05)? | Yes | Yes | Yes | No | No | No | No |
| P value summary | ns | ns | ns | **** | **** | **** | ** |
