# Supplementary material for: Genome-wide association studies meta-analysis uncovers NOJO and SGS3 novel genes involved in Arabidopsis thaliana primary root development and plasticity
Source: Mol Biol Rep. 2024 Jun 14;51(1):763. doi: 10.1007/s11033-024-09623-1 (PMC11178574; doi:10.1007/s11033-024-09623-1)
Supplement: Supplementary file 9 — Supplementary Material 9 [file 11033_2024_9623_MOESM9_ESM.docx]

**Table S9. Pairwise GWAS in inter and intra-studies with high correlation**

| **Pairwise comparison** | **Correlation level** | **Number of SNP shared** | **SNPS with the highest score (-log(P))** | **Score in both studies** | **Gene associated with the SNP with the highest score** |
| --- | --- | --- | --- | --- | --- |
| **Inter-study** | | | | | |
| D2_E4 | 0.98 | 160 | 24810980 | 5.30, 4.84 | AT1G66490 F-box and associated interaction domains-containing protein |
|  |  |  | 24811500 | 5.27, 4.81 | AT1G66500 Pre-mRNA cleavage complex II |
| D3_E5 | 0.96 | 147 | 18054594 | 6.06, 5.77 | AT4G38620. MYB DOMAIN PROTEIN 4 |
|  |  |  | 24810980 | 5.62, 5.03 | AT1G66490 F-box and associated interaction domains-containing protein |
| D4_E5 | 0.9 | 89 | 1215701 | 5.54, 4.32 |  |
|  |  |  | 18054594 | 5.38, 5.77 | AT4G38620. MYB DOMAIN PROTEIN 4 |
| **Intra-study** | | | | | |
| D2_D3 | 0.91 | 94 | 24810980 | 5.30, 5.62 | AT1G66490 F-box and associated interaction domains-containing protein |
|  |  |  | 18054594 | 4.44, 6.06 | AT4G38620. MYB DOMAIN PROTEIN 4 |
|  |  |  | 24811500 | 5.27, 5.13 | AT1G66500 Pre-mRNA cleavage complex II |
| D3_D4 | 0.94 | 104 | 18054594 | 6.06, 5.38 | AT4G38620. MYB DOMAIN PROTEIN 4 |
|  |  |  | 24810980 | 5.62, 4.58 | AT1G66490 F-box and associated interaction domains-containing protein |
|  |  |  | 1215701 | 4.60, 5.54 |  |
| D4_D5 | 0.94 | 100 | 18054594 | 5.38, 5.77 | AT4G38620. MYB DOMAIN PROTEIN 4 |
|  |  |  | 1215701 | 5.54, 4.32 |  |
| D5_D6 | 0.94 | 102 | 1215616 | 6.02, 6.63 | AT4G02735 F-box SKIP17-like protein |
|  |  |  | 1215701 | 5.99, 6.72 |  |
|  |  |  | 7947758 | 5.77, 5.17 | AT5G23575 Transmembrane CLPTM1 family protein |
| D6_D7 | 0.97 | 128 | 1215701 | 6.72, 7.11 |  |
|  |  |  | 1215616 | 6.63, 7.23 | AT4G02735 F-box SKIP17-like protein |
|  |  |  | 8004085 | 6.23, 6.39 |  |
|  |  |  | 12662987 | 5.48, 6.91 |  |
| F4_F5 | 0.93 | 80 | 21013761 | 5.08, 4 |  |
| F5_F6 | 0.96 | 107 | 9215410 | 4.74, 5.23 |  |
| F6_F7 | 0.97 | 130 | 5426133 | 5.16, 5.75 | AT1G15760 Sterile alpha motif (SAM) domain-containing protein |
| F7_F8 | 0.98 | 144 | 5426133 | 5.75, 6.05 |  |
| F8_F10 | 0.97 | 111 | 5426133 | 6.05, 5.24 |  |
| F10_F13 | 0.94 | 84 | 8573982 | 5.62, 5.69 | AT3G23780 Nuclear RNA Polymerase D2A |
