# Supplementary material for: Genome-wide association studies meta-analysis uncovers NOJO and SGS3 novel genes involved in Arabidopsis thaliana primary root development and plasticity
Source: Mol Biol Rep. 2024 Jun 14;51(1):763. doi: 10.1007/s11033-024-09623-1 (PMC11178574; doi:10.1007/s11033-024-09623-1)
Supplement: Supplementary file 11 — Supplementary Material 11 [file 11033_2024_9623_MOESM11_ESM.docx]

**S11 Table**. Pairwise GWAS between studies that shared the same accessions. The top 0.1% of common SNPs are indicated.

| **Pairwise analysis** | **Number of accessions shared** | **Level of correlation** | **Number of SNPs shared** | **SNPs with the highest -log(P)** | **-log_10_(P) in both studies** | **Chr** | **Gene associated** |
| --- | --- | --- | --- | --- | --- | --- | --- |
| **B8_C10** | 107 | 0.458 | 8 | 3455993 | 4.40, 5.77  4.40, 5.77 | 5 | AT5G10946 Hypothetical Protein |
| **C10_E5** | 121 | 0.473 | 1 | 8004085 | 3.14, 3.66 | 5 | - |
| **C10_F3** | 108 | 0.1 | 4 | 19347877 | 3.43, 4.59 | 3 | AT3G52170 DNA Binding protein |
| **D1_F4** | 132 | 0.664 | 13 | 6550883 | 6.10, 4.13 | 2 | - |
| **D2_F5** | 132 | 0.727 | 25 | 11435245 | 4.70, 4.09 | 1 | AT1G31860 Histidine biosynthesis 2 |
| **D3_F5** | 132 | 0.725 | 26 | 11435245 | 3.82, 4.70 | 1 | AT1G31860 Histidine biosynthesis 2 |
| **D3_G5** | 129 | 0.421 | 9 | 1260826 | 3.98, 4.77 | 4 | AT4G02820 Pentatricopeptide repeat (PPR) superfamily protein |
| **E5_F7** | 132 | 0.75 | 16 | 11435245 | 3.84, 5.20 | 1 | AT1G31860 Histidine biosynthesis 2 |
| **E5_G5** | 128 | 0.437 | 4 | 1260826 | 4.36, 5.44 | 4 | AT4G02820 Pentatricopeptide repeat (PPR) superfamily protein |
| **F4_I9** | 109 | 0.159 | 0 | - | - | - | - |
