# Supplementary material for: Genome-wide association studies meta-analysis uncovers NOJO and SGS3 novel genes involved in Arabidopsis thaliana primary root development and plasticity
Source: Mol Biol Rep. 2024 Jun 14;51(1):763. doi: 10.1007/s11033-024-09623-1 (PMC11178574; doi:10.1007/s11033-024-09623-1)
Supplement: Supplementary file 12 — Supplementary Material 12 [file 11033_2024_9623_MOESM12_ESM.docx]

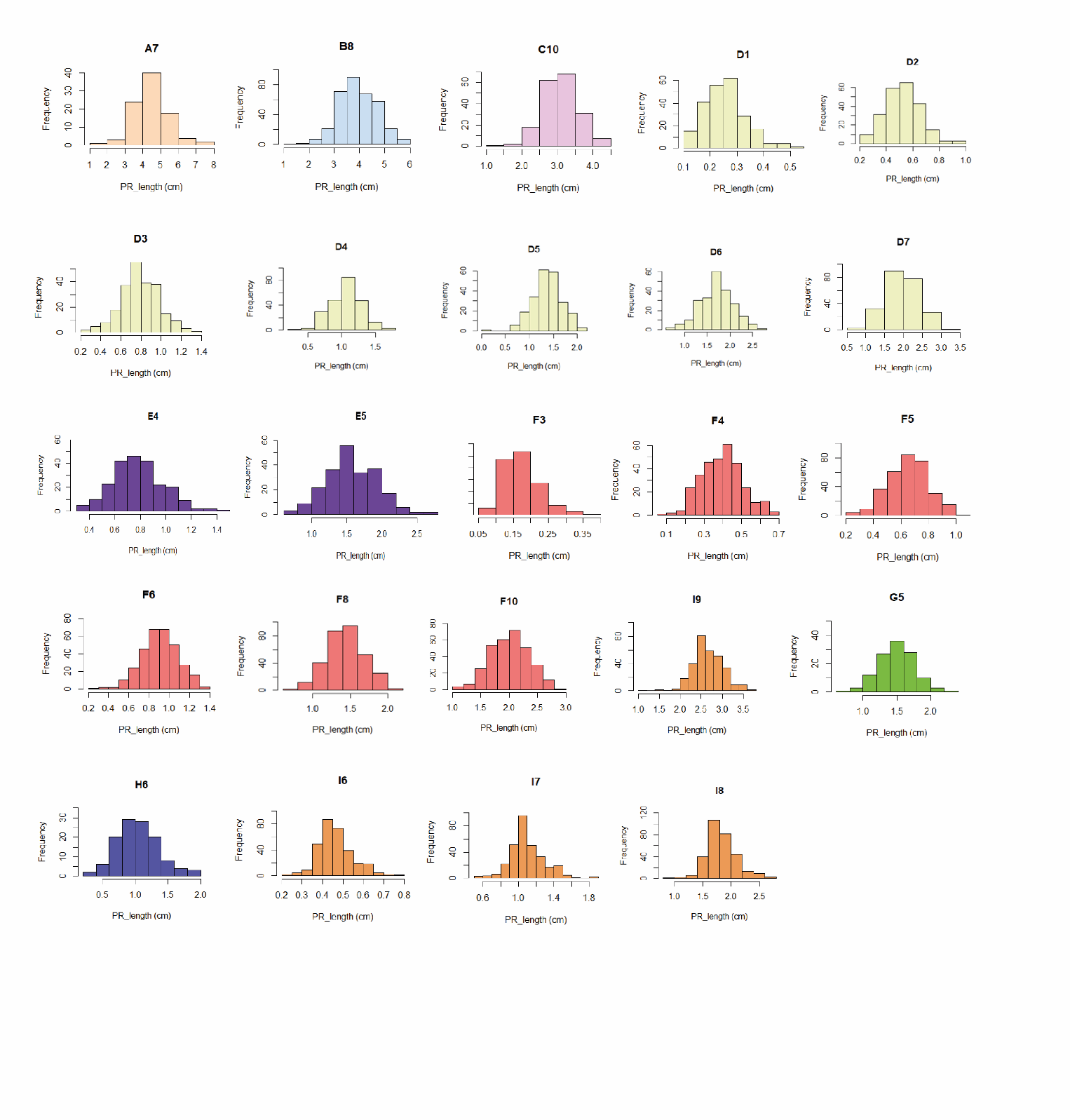


**S1 Fig. Histograms of the published data.** The letter corresponds to each study, and the number means the DAG evaluated. Lachowiec et al., 2015 (A7), Julkowska et al., 2017 (B8), Ristova et al., 2018 (C10), Bouain, et al., 2018 (D1-D7). Bouain, et al., 2019 (E4-E5), Li et al., 2019 (F3-F8, F10, F13); Ogura et al., 2019 (G5), Justamante et al., 2019 (H6), Deolu-Ajayi et al., 2019 (I6-I9)


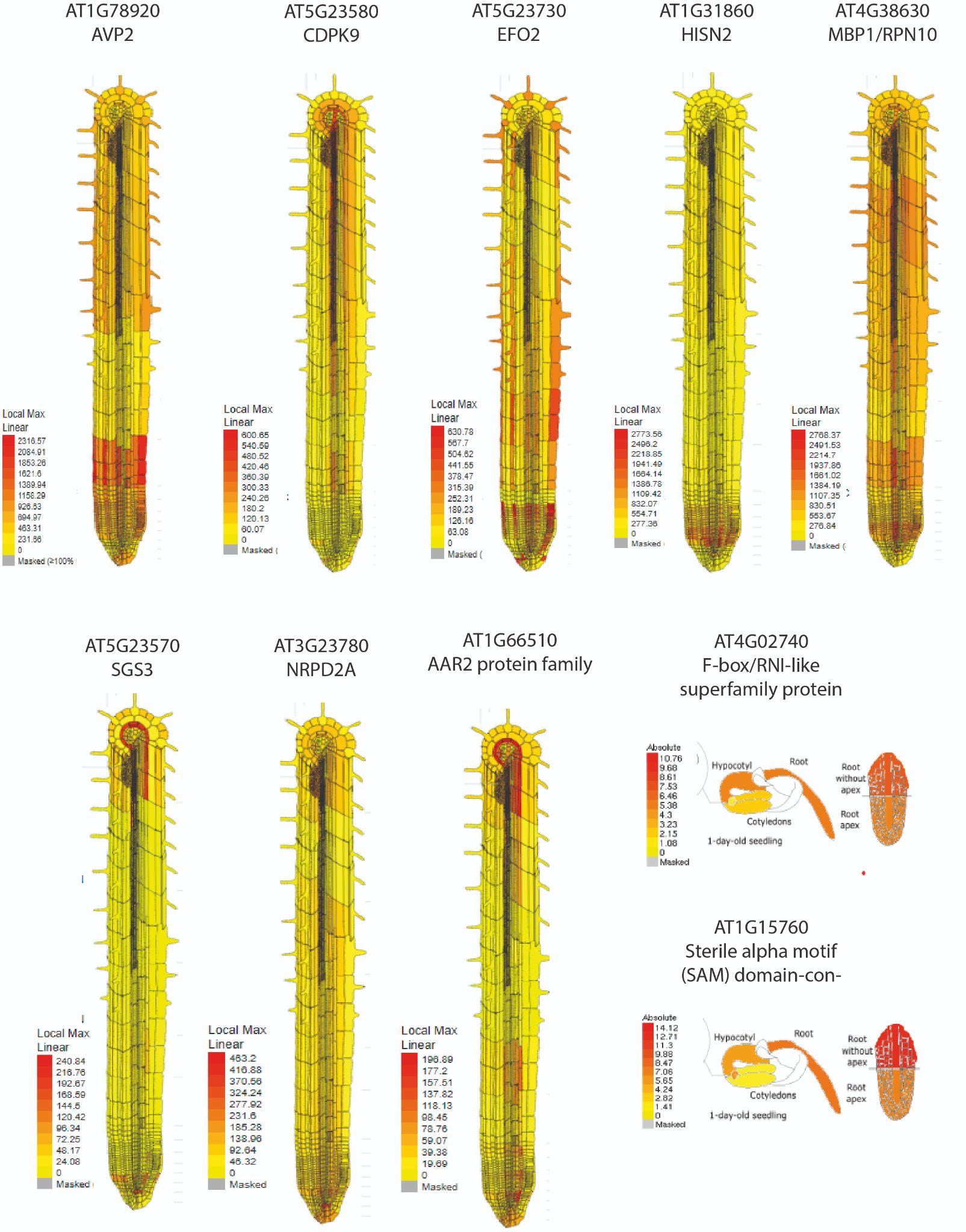


**S2 Fig.** Root expression of candidates genes found in GWAS. The images were retrieved from the Plant and Tissue and Experiment eFP Viewer (<http://bar.utoronto.ca/eplant/>)


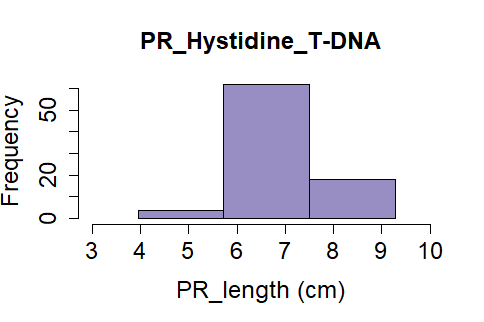


**S3 Fig.** PR length division of *his2* heterozygous line into short, medium and long PR according to Sturge’s rule.


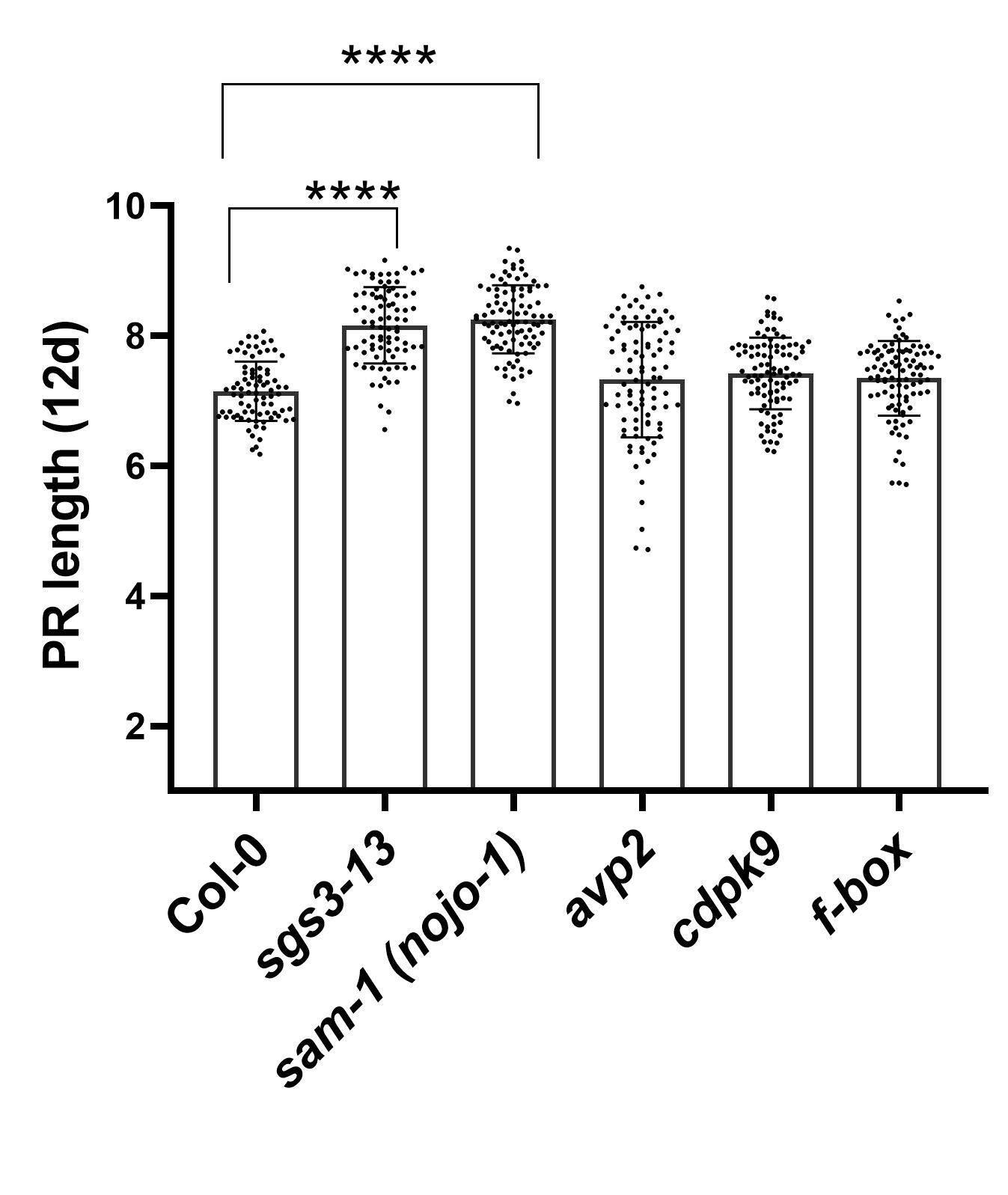


**S4 Fig.** 12 days PR length of T-DNA lines from candidate genes obtained from GWAS


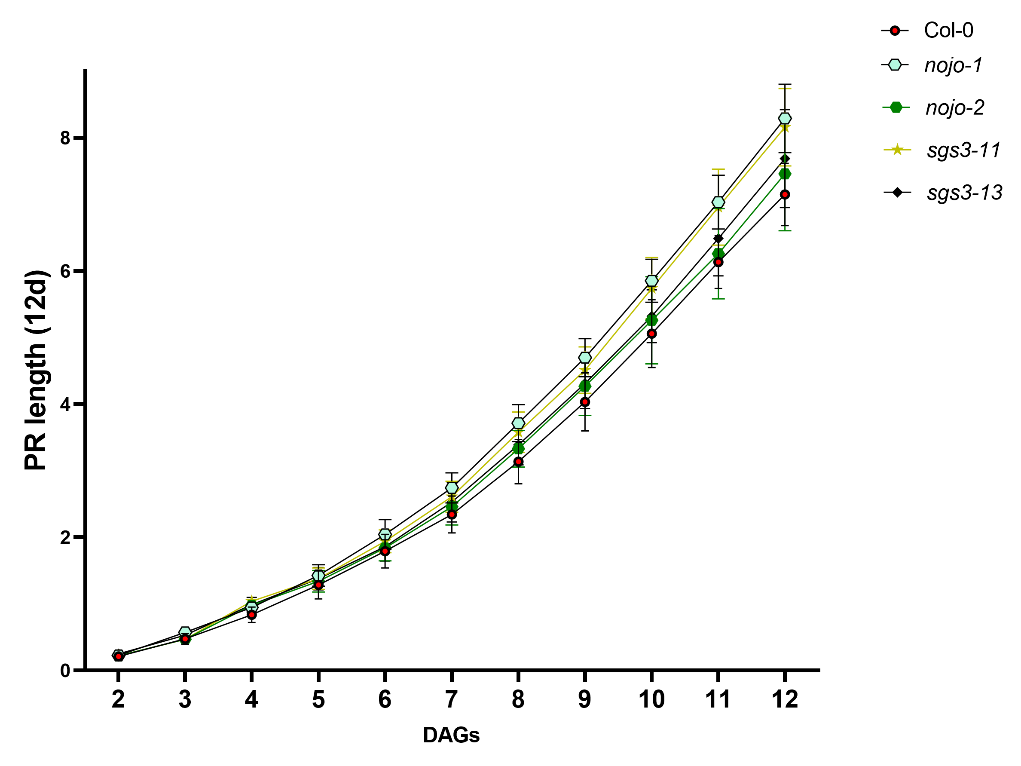


**S5 Fig.** PR length kinetics over 2- 12 days of *nojo-1, nojo-2, sgs3-11* and *sgs3-13*.


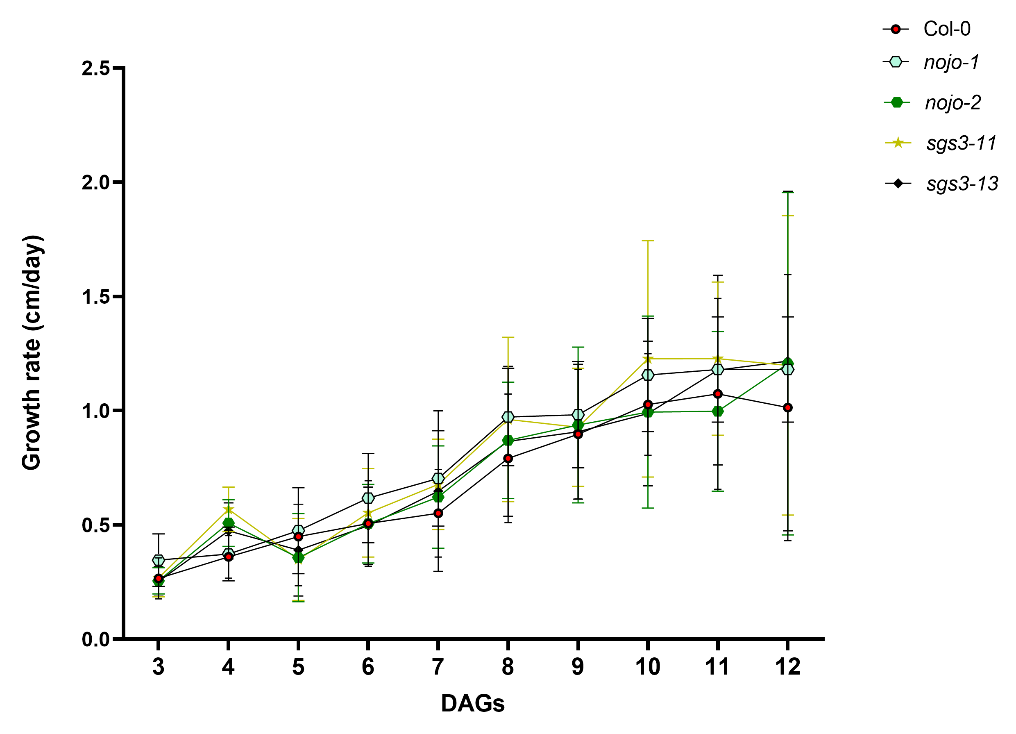


**S6 Fig.** Growth rate over 2-12 days of *nojo-1, nojo-2, sgs3-11* and *sgs3-13.*
